# Knowledge, Attitude, and Practice of Healthcare Professionals and Medical Students Regarding Probiotics and Prebiotics in Lahore, Pakistan: A Cross-Sectional Study

**DOI:** 10.7759/cureus.61788

**Published:** 2024-06-06

**Authors:** Fatima Khalid, Hira Aamer, Huda Tarique, Mehreen Yawar, Maha Tariq, Muhammad Shaheryar, Abdul Haseeb Hasan

**Affiliations:** 1 Community Medicine, Shalamar Medical and Dental College, Lahore, PAK; 2 Internal Medicine, Mayo Hospital, Lahore, PAK

**Keywords:** knowledge attitude practices studies, community medicine, public health care, epidemiology and public health, prebiotics, efficacy of probiotics

## Abstract

Objective

This study aims to explore healthcare professionals’ and medical students’ knowledge and attitudes toward probiotics and prebiotics in various health conditions. It seeks to identify any obstacles associated with their use and gain insight into the healthcare community’s perspectives on these supplements.

Methods

A descriptive cross-sectional study was conducted using a preformed questionnaire. Data was collected by a convenience sampling technique during October and November 2023. A total of 417 responses were collected, and the data analysis was performed using IBM SPSS Statistics for Windows, Version 20.0 (Released 2011; IBM Corp., Armonk, NY, USA).

Results

In the study, 198 participants (47.5%) were doctors, and 219 (52.5%) were medical students. Only 81 (37%) students had good knowledge about probiotics, while 36 (16.4%) had good knowledge about prebiotics. Poor knowledge was associated with a poor knowledge, attitude, and practice (KAP) score, indicating a link between knowledge, attitude, and practice. Similarly, only 96 (48.5%) doctors had good knowledge about probiotics, while 45 (22.7%) of them had good knowledge about prebiotics. The study found that a lack of knowledge was the primary barrier to the use of prebiotics and probiotics, as reported by 226 (54.4%) participants. The chi-square test showed no significant correlation between participants’ demographics and their KAP.

Conclusion

The majority of respondents demonstrated poor knowledge and practices regarding probiotics and prebiotics, which can be attributed to insufficient awareness of their benefits. Education tools like curriculum and training programs should include evidence-based information to raise awareness among healthcare professionals about their benefits and address concerns associated with their use in treating patients.

## Introduction

The term “probiotic” originates from the Latin preposition “pro,” signifying “for,” and the Greek adjective “bios,” meaning “life,” first coined in the 1960s [1]. WHO and the Food and Agriculture Organization of the United Nations define probiotics as “live microorganisms that, when administered in adequate amounts, confer a health benefit on the host” [2,3]. While commonly found in fermented foods like yogurts, probiotic supplements have evolved to include formulations in tablets, capsules, granules, and liquids, with specific emphasis on strains from *Lactobacillus *and *Bifidobacterium* species [4].

Conversely, prebiotics represent substrates assimilated by the gut microbiota to enhance health [5]. According to the Food and Agriculture Organization of the United Nations, prebiotics are “nonviable food components that confer a health benefit on the host associated with the modulation of the microbiota” [6]. Widely recognized prebiotics, such as fructans, inulin, fructooligosaccharides, and galactooligosaccharides, are generally regarded as safe [7,8]. Synbiotics, a fusion of probiotics and prebiotics, typically feature combinations of *Bifidobacterium *or *Lactobacillus *bacteria with fructooligosaccharides, with the latter being particularly popular [9].

The past two decades have witnessed a surge in the popularity of probiotics and prebiotics due to a continuously expanding body of scientific evidence supporting their positive impact on human health [10-12]. Probiotics primarily exert their effects in the colon, the major habitat of bodily bacteria, employing diverse mechanisms, including the inhibition of gut pathogens [13], beneficial modulation of colonic metabolism [14], and stimulation of immune responses [15]. Extensive research attests to the effectiveness of probiotics in addressing conditions such as lactose intolerance, acute gastroenteritis, antibiotic-associated diarrhea, inflammatory bowel disease (IBD), necrotizing enterocolitis, allergies, dental caries, *Helicobacter pylori *eradication, hypercholesterolemia, and cancer [16].

Moreover, prebiotics offer valuable support either as an alternative or in conjunction with probiotics, with documented benefits in colon cancer, IBD, cardiovascular disease, glycemic control in type 2 diabetes, and weight management [17]. The combined use of probiotics and prebiotics as synbiotics presents further evidence of health benefits [17,18]. Despite these advantages, awareness among healthcare practitioners remains insufficient. Studies, including one conducted in Jordan [19] and another in Pakistan [20], reveal a lack of knowledge, with only 51.6% and 15.1% of healthcare workers demonstrating awareness of probiotics, respectively.

The escalating production of probiotics and prebiotics, fueled by recent robust research supporting their beneficial effects, underscores the crucial need for heightened awareness among healthcare professionals and medical students. Notably, developed countries like Australia [21], Canada [22], and the United States [23] exhibit a high level of awareness, knowledge, and consumption of probiotic supplements, a contrast evident in developing nations such as Pakistan. A dearth of information on probiotics in Pakistan, as revealed by literature searches, emphasizes the necessity of investigating the awareness and knowledge levels of healthcare professionals and medical students. Recognizing the significant benefits probiotics may offer in treating medical conditions, this study seeks to assess the knowledge, aptitude, and practices of healthcare professionals and medical students concerning probiotics and prebiotics in the city of Lahore, Pakistan.

## Materials and methods

Study design

A descriptive cross-sectional study was carried out in Lahore, Pakistan to evaluate the knowledge, attitude, and practice (KAP) of healthcare professionals and students regarding prebiotics and probiotics. Additionally, the study aimed to identify the barriers to their usage. A preformed questionnaire was used as the tool to collect data, and a convenience sampling technique was employed to gather responses during October and November 2023. The subjects of the study included registered doctors in Lahore and medical students enrolled in MBBS. The questionnaire contained an informed consent statement at the beginning, and incomplete responses were excluded from the final results.

Sample size

A sample size of 377 was calculated using the online sample size calculator Raosoft (Raosoft Inc., Seattle, United States), assuming a 95% CI, a response rate of 50%, a Z of 1.96, and a margin of error of 5%. The calculated sample size was increased by 10% to minimize any errors that may occur; hence, the final sample size was 417.

Questionnaire development

We used the questionnaire from a KAP study carried out by Arshad et al. [20] in Multan, Pakistan, as a reference for our questionnaire. While their questionnaire evaluated the KAP for probiotics only, we derived similar questions from it for prebiotics as well. The questionnaire was found to be reliable, with a Cronbach’s alpha value of 0.846. A total of 14 figures, depicting the questionnaire utilized in this research, are provided in the appendix for reference. These figures serve to illustrate the structure and content of the questionnaire used for data collection. A supplemental table containing the scoring pattern used for good and poor scores has also been uploaded. The Google Form of the questionnaire was distributed among medical students and healthcare professionals through various forms of social media.

The final questionnaire consisted of three sections. The first section collected demographic data such as gender, age, marital status, professional position, patient population, and years of practice of the participants. The second section contained questions related to probiotics, while the third section contained questions related to prebiotics.

The section on probiotics covered several topics, including knowledge about probiotics, popular brands in Pakistan, attitudes toward using probiotics, current usage practices, and obstacles to using them.

The first question asked participants to define probiotics. The second question used a Likert scale (0 = not at all, 1 = somewhat, and 2 = very much) to assess how well-known different commercial probiotic brands are in Pakistan. The third question tested knowledge about 10 diseases or health conditions where probiotics are beneficial, also using the Likert scale. Other questions were asked about specific bacterial strains that are considered probiotics and the sources from which participants got their information about probiotics.

The attitude of participants toward probiotics was also assessed using the Likert scale, using the inverted score for a negative question. Ten health conditions were used to assess the participants’ practices regarding probiotics, and the Likert scale was again used. Finally, the last subsection determined the barriers to probiotic use among the participants, where they could choose multiple options for the reasons why they do not recommend probiotics to patients.

The third section had similar questions about the knowledge of prebiotics, including their definition, conditions in which they might be beneficial, food sources of prebiotics, and sources of information regarding prebiotics. The attitude and practice regarding prebiotics were assessed in exactly the same way as for probiotics, with the incorporation of the Likert scale. The last question explored the barriers to their use.

Data collection

The questionnaire on Google Forms was distributed to the eligible participants online through email and various forms of social media platforms. A total of 417 responses were collected. The questionnaire accepted responses from October 2023 to November 2023.

Statistical analysis

In our study, the data analysis was performed using IBM SPSS Statistics for Windows, Version 20.0 (Released 2011; IBM Corp., Armonk, NY, USA). The frequencies and percentages of categorical variables were used in the descriptive statistics. The chi-square test was used to analyze the KAP between the demographic subgroups. A p-value of <0.05 was considered to be statistically significant.

## Results

The study gathered and analyzed a total of 417 responses from participants who were asked questions to evaluate their knowledge about probiotics and prebiotics. A total of 49.2% (n = 206) of the participants were males, while around 50.8% (n = 211) were females. In terms of occupation, approximately 47.5% (n = 198) were doctors, and 52.5% (n = 219) were medical students. Among the doctors, the majority (n = 145, 73.2%) had one to four years of work experience, and 69.7% (n = 138) of them dealt with the adult population. More detailed information about the demographic characteristics of the participants is presented in Table 1.

**Table 1:** Demographic characteristics of the participants

Statistical demographic characteristics	Total (n = 417), N	Percentage, %
Gender		
Male	206	49.2
Female	211	50.8
Age (mean +/- SD = 24.61 +/- 3.682)		
<20	8	1.9
21-25	302	73.2
26-60	104	24.8
Marital status		
Married	66	15.9
Unmarried	351	84.1
Professional position		
Medical student	219	52.2
Doctor	198	47.5
Patient population		
Pediatrics	9	4.5
Adults	138	69.7
Geriatrics	1	0.5
Pediatrics and adult	9	4.5
Adult and geriatrics	23	11.6
Pediatrics, adult, and geriatrics	18	9.1
Workplace experience		
Student	217	52.2
1-4 years	162	39
>5 years	36	8.6

It is interesting to note that 76.1% (n = 317) of the respondents were able to correctly describe the definition of probiotics, while only 62.7% (n = 260) were able to correctly identify the definition of prebiotics. Out of all the medical students, only 37% (n = 81) demonstrated good knowledge about probiotics. Among the 138 students who had a poor knowledge score on probiotics, 128 also had a poor overall KAP score. When it came to prebiotics, only 16.4% (n = 36) of the medical students showed good knowledge. Among the 170 students with a poor overall KAP score, 160 of them also had a poor knowledge score. This highlights the strong connection between KAP. Furthermore, there was no statistical significance found between gender, age, or marital status.

The effect of demographic characters on medical students’ KAP scores is illustrated in Table 2.

**Table 2:** Effect of demographic characters on medical students’ KAP scores KAP, knowledge, attitude, and practice

Variables	% with poor scores	% with good scores	p-value
Probiotics			
Gender			0.796
Female	73.5	26.5	
Male	71.9	28.1	
Age			0.396
19 years	80	20	
20 years	100	0	
21 years	70	30	
22 years	69.2	30.8	
23 years	75.5	24.5	
24 years	71.4	28.6	
25 years	80	20	
26 years	0	100	
Marital status			0.708
Unmarried	72.4	27.6	
Married	80	20	
Prebiotics			
Gender			0.762
Female	78.6	21.4	
Male	76.9	23.1	
Age			0.142
19 years	80	20	
20 years	100	0	
21 years	80	20	
22 years	70.8	29.2	
23 years	80.9	19.1	
24 years	81	19	
25 years	90	10	
26 years	0	100	
Marital status			0.339
Unmarried	78	22	
Married	60	40	

Only 48.5% (n = 96) of doctors had good knowledge about probiotics, and just 22.7% (n = 45) had good knowledge of prebiotics. Interestingly, the study found that males (n = 236, 56.5%) had better knowledge than females (n = 177, 42.5%), which differed from previous studies. However, no significant associations were found with age, marital status, experience, or patient population. The effect of demographic characters on doctors’ KAP scores is illustrated in Table 3.

**Table 3:** Effect of demographic characters on doctors’ KAP scores KAP, knowledge, attitude, and practice

Variables	% with poor scores	% with good scores	Mean ± SD	p-value
Probiotics				
Age				0.51
25 years and below	71.9	28.1	22.42 ± 8.38	
25 years to 60 years	67.6	32.4	23.16 ± 7.29	
Experience				0.22
4 years or less	71.6	28.39	22.35 ± 7.55	
5 years or more	61.11	38.88	24.83 ± 8.79	
Marital status				0.61
Unmarried	70.8	29.2	22.50 ± 7.59	
Married	67.2	32.8	23.49 ± 7.55	
Prebiotics				
Age				0.45
25 years and below	67.4	32.6	23.68 ± 9.25	
25 years to 60 years	72.3	27.7	24.68 ± 7.20	
Experience				0.36
4 years or less	71.6	28.39	23.81 ± 8.12	
5 years or more	63.88	36.12	25.94 ± 8.70	
Marital status				0.22
Unmarried	72.99	27	23.45 ± 8.48	
Married	63.93	36.06	25.88 ± 7.51	

**Figure 1:**
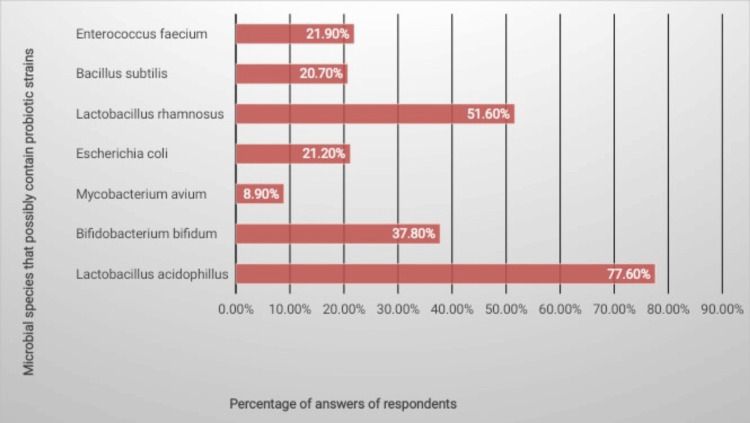
Percentages of various microbial species having probiotic strains as per the knowledge of participants

A total of 37.9% (n = 158) of the respondents were very familiar with the drug brand Enflor sachets and capsules. When asked to select species with probiotic strains, the most recognizable ones were *Lactobacillus acidophilus *(n = 322, 77.60%) and *Lactobacillus rhamnosus *(n = 214, 1.60%). Some people also chose *Enterococcus faecium *(n = 91, 21.90%), *Bifidobacterium bifidum *(n = 157, 37.80%), and *Escherichia coli *(n = 88, 21.20%). However, it is worth mentioning that a small percentage of respondents (n = 37, 8.90%) incorrectly selected *Mycobacterium avium*. Percentages of various microbial species having probiotic strains as per the knowledge of participants are shown in Figure 1.

For certain questions, participants could choose from three response options: “not at all,” “somewhat,” or “very much.” This allowed them to indicate their level of agreement or interest. According to the survey, more than half of the respondents, around 57.5% (n = 240), believed that probiotics are very beneficial for general gut health. On the other hand, only 38.1% (n = 159) considered prebiotics to be beneficial for general gut health. When it comes to specific conditions, such as acute diarrhea and antibiotic-associated diarrhea, over half of the respondents (n = 224, 53.7%) were in favor of giving probiotics, while only 39.1% (n = 163) believed that prebiotics were very beneficial in antibiotic-associated diarrhea. For irritable bowel syndrome (IBS), about half of the respondents found both probiotics and prebiotics to be somewhat helpful. Additionally, 51.8% (n = 216) believed that probiotics are somewhat important when dealing with IBD, while 48.9% (n = 204) believed the same for prebiotics. It is interesting to note that nearly half of the respondents (n = 200, 48%) believed that prebiotics need to be alive organisms, and 46% (n = 192) believed that taking prebiotics for a long period of time is necessary as they disappear from the gut after two weeks. Lastly, around 48% (n = 200) of the respondents considered garlic and apples as naturally occurring sources of prebiotics.

The majority of respondents obtained information about probiotics from websites (n = 200, 48%) and curriculum books (42%, n = 175). Similarly, for prebiotics, 47.2% (n = 196) turn to websites for information, while 39.8% (n = 165) rely on curriculum books. Regarding probiotics, 56.1% (n = 235) considered them good, 55.6% (n = 231) were sure that they were not detrimental to health, and 57.1% (n = 238) showed willingness to prescribe them when provided with proper reference.

A total of 49.3% (n = 108) of medical students showed a good attitude toward probiotics. Among the 111 (50.7%) students who had poor attitude scores, 88 of them had poor overall KAP scores, identifying the role of knowledge associated with attitudes. Doctors who displayed good attitudes toward probiotics were 58.6% (n = 116). Moreover, 59.3% (n = 67) of females and 57.6% (n = 49) of the male population showed good attitudes pertaining to no significant association with gender, unlike previous studies [19]. A total of 63% (n = 138) of medical students showed a positive attitude toward prebiotics. Out of the 81 (37%) who had poor attitudes, 72 showed poor overall KAP scores. Furthermore, 83.3% (n = 165) of doctors had positive attitudes toward prebiotics. Again, statistical significance could not be established.

More than half the population (53.4%; n = 217) thought that prebiotics were not at all bad for health. A total of 52.2% (n = 222) considered them somewhat good, and 48.1% (n = 202) were keen on recommending them.

Participants were asked about their practice of recommending probiotics and prebiotics for health conditions including general gut health, acute diarrhea or antibiotic-associated diarrhea, IBS, IBD, heart health, mental health, *Clostridium difficile* infections, elderly patients with comorbid conditions, prevention of respiratory or urinary tract infections, obesity, and allergies, with one of three possible answers stating not at all, somewhat, and very much.

Good practice is based on whether the participants recommend and prescribe probiotics and prebiotics to their patients for medical conditions where they have been proven to be beneficial through research. A total of 19.2% (n = 42) of medical students demonstrated good practice with probiotics. Among the 177 students who had poor practice scores, 155 of them had poor overall KAP scores regarding probiotics. Moreover, 41.7% (n = 91) of medical students showed good practice toward prebiotics. All the 125 students who had poor practice scores were part of those 170 participants who had poor overall KAP scores regarding prebiotics. Doctors who displayed good practice toward probiotics were 11.6% (n = 23), while those who displayed good practice toward prebiotics were 29.8% (n = 59).

In the study, around 30.6% (n = 127) of the participants strongly supported recommending probiotics for overall gut health, while only 28.6% (n = 119) recommended prebiotics for general health. For acute diarrhea and antibiotic-associated diarrhea, 38.3% (n = 159) prescribed probiotics, and 32.7% (n = 136) prescribed prebiotics. When it comes to IBS, approximately 40% (n = 166) of the respondents somewhat recommended both probiotics and prebiotics. Similarly, for IBD, almost 35% (n = 146) of the participants somewhat recommended both probiotics and prebiotics. In terms of* C. difficile *infection, 32.2% (n = 134) of the respondents somewhat considered prescribing probiotics, while 38.3% (n = 159) somewhat prescribed prebiotics for elderly individuals with comorbid conditions. In the study, there were fewer recommendations for probiotics or prebiotics specifically for heart health, mental health, obesity, or allergies. It seems like probiotics and prebiotics are considered useful mainly for gut health-related conditions.

**Figure 2:**
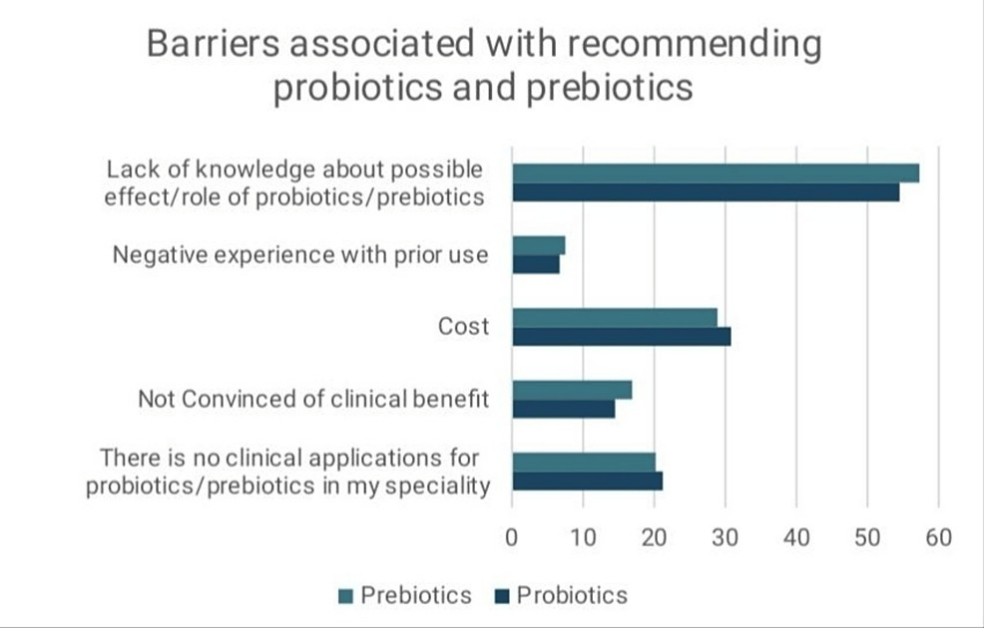
Barriers associated with recommending probiotics and prebiotics (percentage %)

Participants were allowed to choose multiple options regarding barriers. The leading cause was a lack of knowledge (n = 226, 54.4%). Cost concerns and the perception that these products had no use in particular specialties were other common causes. The rest of the causes are displayed in Figure 2.

## Discussion

Statement of principal findings

This study evaluated the KAP of healthcare professionals and medical students regarding probiotics and prebiotics. The results showed that a considerable number of respondents had a clear understanding of the definition of probiotics and prebiotics. However, their knowledge about specific strains and practices related to probiotics and prebiotics was inadequate. Despite this, the respondents had a positive attitude toward their application. The survey indicated that most healthcare professionals and medical students relied on websites for information on probiotics and prebiotics. However, the actual prescription of probiotics and prebiotics was limited due to concerns about cost and clinical applications. The study did not find any significant correlation between the KAP of the respondents and their demographic characteristics, as determined by a chi-square test. This study emphasizes the need for enhanced education and awareness among healthcare professionals to ensure the safe and effective use of probiotics and prebiotics.

Strengths and weaknesses of the study

The study sheds light on an area of research that has not been extensively studied in Pakistan, providing valuable insights. The sample size was increased to minimize errors and enhance the reliability of the findings. This study offers a comprehensive analysis of KAP regarding probiotics and prebiotics. However, it does have a few limitations. The small sample size and the use of a convenience sampling technique may introduce bias, which could limit the generalizability of the results. It should be noted that the findings are specific to the city where the study was conducted and may not be representative of the entire country. Lastly, the study did not evaluate the effectiveness of probiotics and prebiotics, which would have been helpful in guiding clinical practice.

Strengths and weaknesses in relation to other studies, discussing important differences in results

While several surveys have been conducted on probiotic use in recent years [19,20,24], our survey stands out as one of the few that evaluates knowledge and understanding of prebiotics among healthcare professionals. Additionally, it involves medical students as well, to gain insight into early-stage medical education. Our study contributes to the limited research on probiotics and prebiotics in Pakistan. We identified significant differences and similarities in our results compared to previous studies that are worth highlighting.

Regarding probiotics, only 56.1% (n = 234) of participants considered them good for health, compared to 87.7% reported in a previous study in India [25]. Unlike other studies, no significant relationship with gender or patient population could be established [19,20]. This highlights the lack of knowledge at all levels and niches and the importance of the dire need to improve awareness. A total of 76.1% (n = 317) of the respondents correctly identified the definition of probiotics. This finding is consistent with an international survey where 81.3% of medical doctors identified the correct definition [24]. In our study, 37% (n = 81) of the medical students and 48.5% (n = 96) of the doctors showed good knowledge about probiotics, which is somewhat consistent with reports from Jordan [19]. These results surpass those of another study conducted in Pakistan, where only a small percentage of healthcare professionals (15.1%) exhibited good knowledge regarding probiotic use, whereas practicing pharmacists in that study demonstrated better knowledge [20].

Our study found that 77.6% (n = 322) of participants correctly identified* L. acidophilus *as a probiotic species. Among the species listed, *M. avium *was the only one lacking a probiotic strain, yet it was selected by 8.9% (n = 37) of the respondents. This percentage is higher compared to the international survey, where only 3.5% of medical doctors incorrectly selected *M. avium *as a probiotic [24].

In our study, almost half of the participants (n = 200, 48%) believed that probiotic bacteria must be alive, compared to 73.7% in the international survey [24]. In Nigeria, only 28.3% of respondents thought that probiotics contain live organisms [26].

When it comes to the timing of probiotic intake, it is worth noting that 35.9% (n = 150) of our respondents asserted that probiotics should be taken before meals, which is not consistent with the international survey, where 64% preferred taking them before meals [24]. It was noted that approximately 42% (n = 175) of the respondents turn to curriculum books for information regarding probiotics. These findings are consistent with an international survey where 53.3% of participants reported getting information primarily from books and expert magazines [24].

It was found that 58.6% (n = 116) of doctors had a positive attitude toward the use of probiotics, which was consistent with a study in Jordan, but the numbers were far less compared to other studies [19,20,25].

Based on our research, approximately 11.6% (n = 23) of doctors demonstrated good practice when it comes to probiotics. This finding is consistent with a similar study conducted in Pakistan [20], where 15.6% of healthcare professionals, including pharmacists, showed good practices. Additionally, an international survey [24] showed that 15.6% of participants from various specialties exhibited good practices. However, it was far less compared to the study in Nigeria, where 35.1% of physicians prescribed the use of probiotics [26].

The available literature related to prebiotics mostly focuses on their sources, properties, and effectiveness in various health conditions [5,7,9,10,27]. A survey was conducted in rural areas of the USA [23] to assess the general population’s attitudes and practices toward the use of dietary supplements, which included some prebiotics like garlic. The survey found that 16.3% of participants preferred using garlic for their health issues. However, no similar survey has been conducted for healthcare professionals or medical students to assess their perceptions regarding prebiotics. Surprisingly, the doctors and medical students in our study had better attitudes and practices toward prebiotics than probiotics. This can be due to the availability of prebiotics in natural forms and people being more receptive to dietary restrictions than to the consumption of pharmaceutical products.

Our study displayed a lack of knowledge as the most common barrier to the use of both probiotics and prebiotics. This was consistent with previous studies conducted on probiotics [19,20].

Variations in KAP compared to studies in other countries suggest potential gaps in education and awareness within Pakistan’s healthcare system. These differences may be due to variations in sample size, geographic location, and the study’s methodology.

Meaning of the study: possible explanations and implications for clinicians

The results of the survey indicate that there is a need to improve healthcare professionals’ understanding of probiotics and prebiotics in Pakistan. To enhance their knowledge, professional medical associations can arrange educational activities such as continuing medical education programs and focused training. Additionally, incorporating evidence-based knowledge into medical curricula and disseminating information through accessible channels like websites and curriculum books can help raise awareness and improve practices in the field. By improving healthcare professionals’ understanding of probiotics, it is possible to ensure the safe and effective use of these products among the general population [20].

Unanswered questions and future research

The study highlights the need for further research on the effectiveness of probiotics and prebiotics for various health conditions. Furthermore, extensive qualitative research can shed light on certain attitudes and practices that emphasize the cultural and contextual factors influencing decision-making among healthcare professionals. This can help in evaluating the best ways to educate and train healthcare professionals on the use of probiotics and prebiotics. Finally, future studies should investigate the potential risks associated with probiotic and prebiotic use, particularly in vulnerable populations, such as immunocompromised patients.

Comparative studies across different regions of Pakistan can provide insights into regional variations and inform targeted interventions. Longitudinal studies tracking changes in knowledge and practices over time can assess the effectiveness of educational initiatives and interventions. Qualitative research can delve deeper into the reasons behind certain attitudes and practices, shedding light on cultural and contextual factors influencing decision-making among healthcare professionals.

## Conclusions

Most respondents demonstrated poor knowledge and practices pertaining to the use of probiotics and prebiotics but a positive attitude toward their application. It can be concluded that poor practices can stem from a lack of knowledge regarding the benefits of probiotics and prebiotics. Moreover, with the advancing research and extensive information regarding the usage of probiotics and prebiotics, it is necessary to incorporate evidence-based knowledge into education tools like curriculum, media, and focused training programs to raise awareness among healthcare professionals regarding probiotics and prebiotics while addressing the reservations and concerns associated with their application in the treatment regimens of patients. Furthermore, similar additional studies should be conducted in order to advance the evidence-based decision-making skills of healthcare professionals.

## Figures and Tables

**Figure 3:**
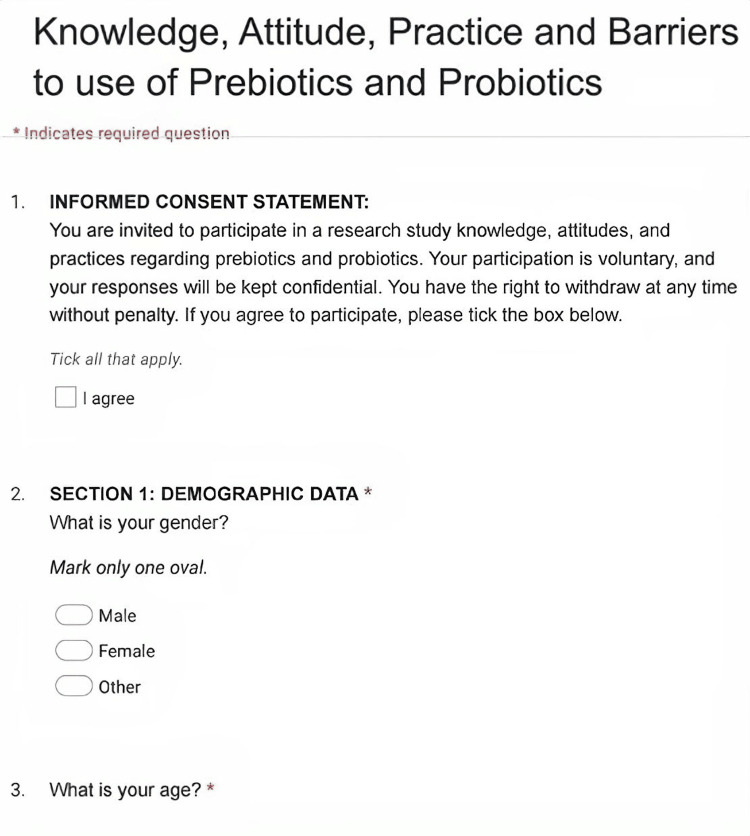
Questionnaire

**Figure 4:**
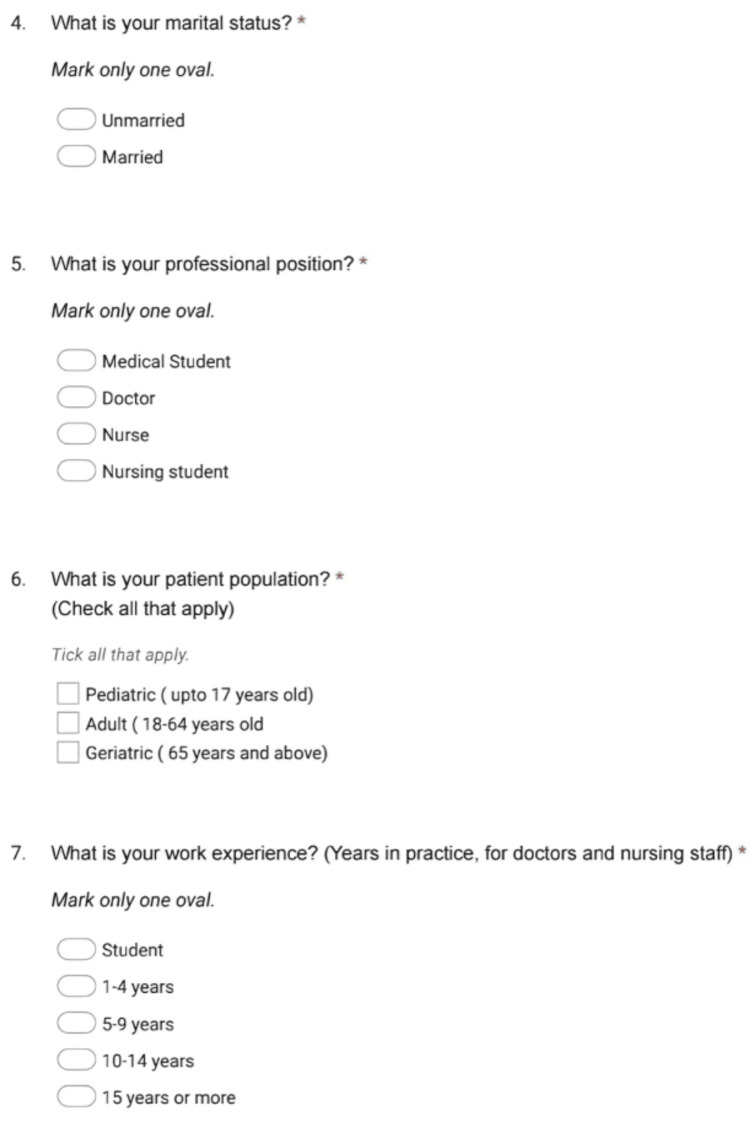
Questionnaire

**Figure 5:**
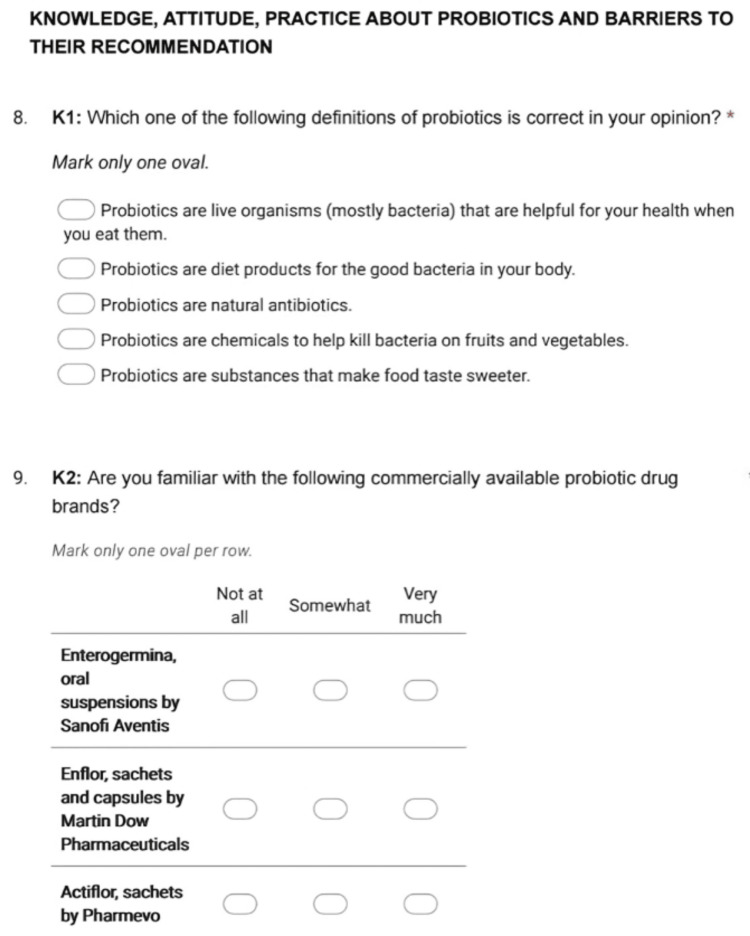
Questionnaire

**Figure 6:**
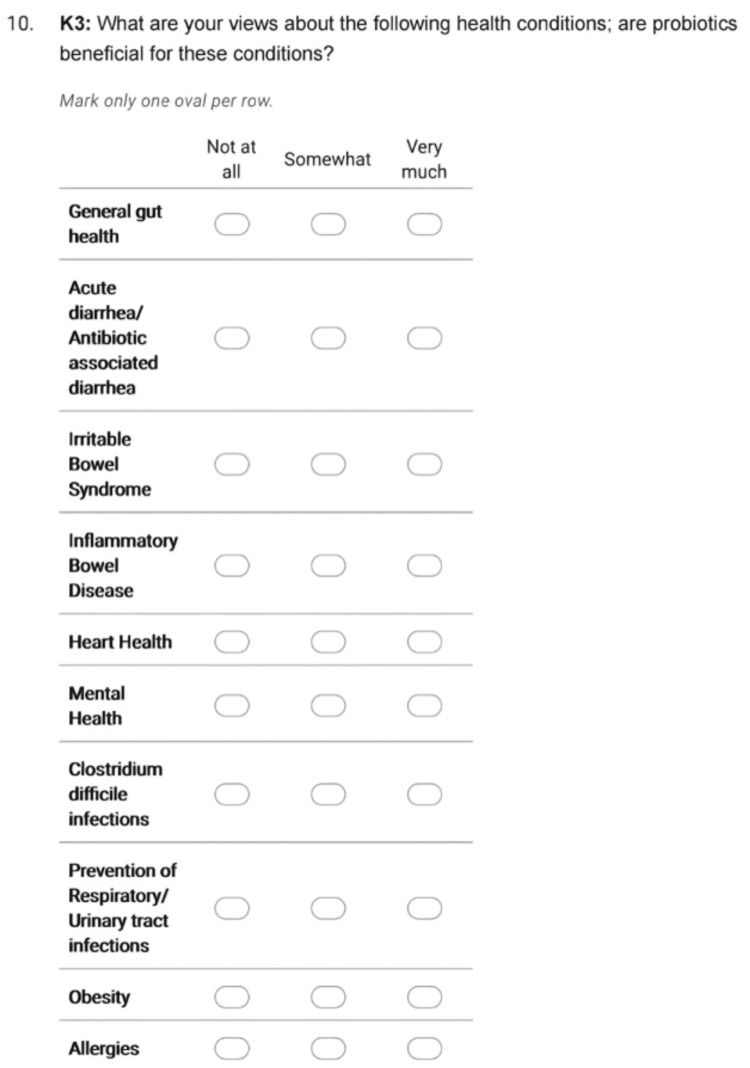
Questionnaire

**Figure 7:**
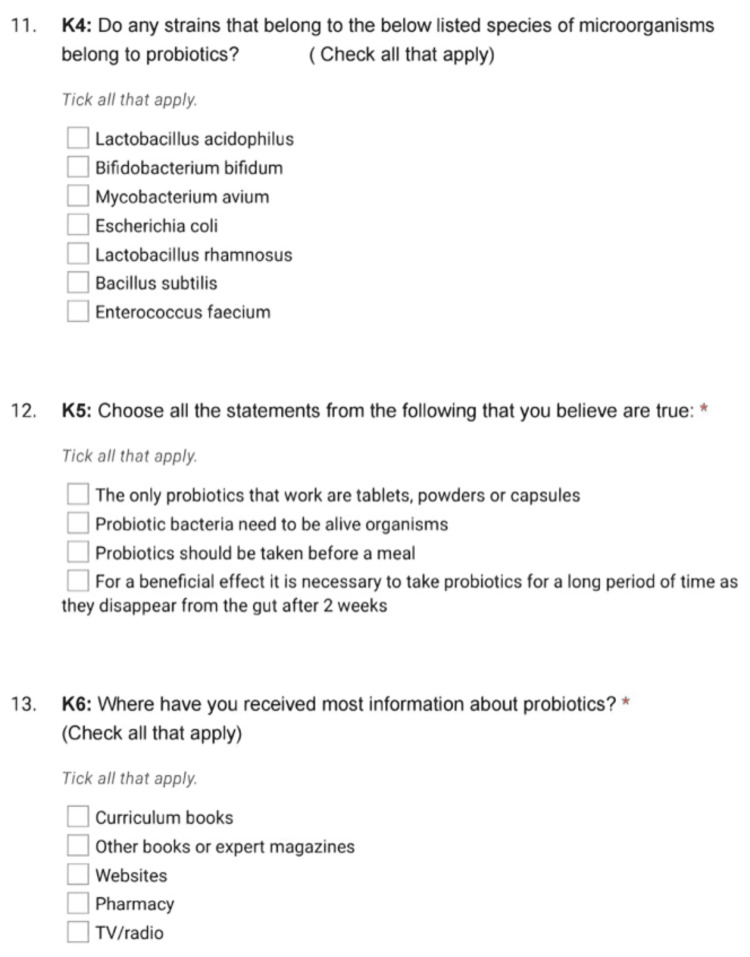
Questionnaire

**Figure 8:**
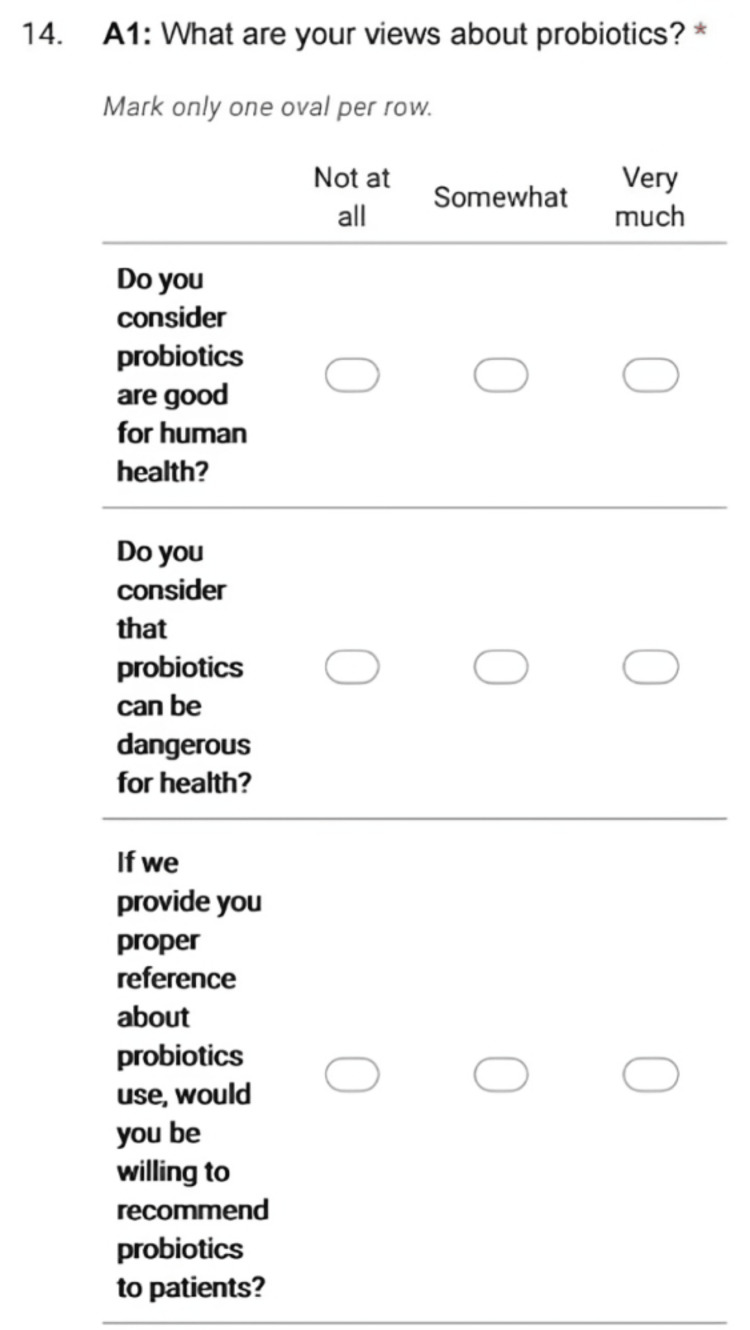
Questionnaire

**Figure 9:**
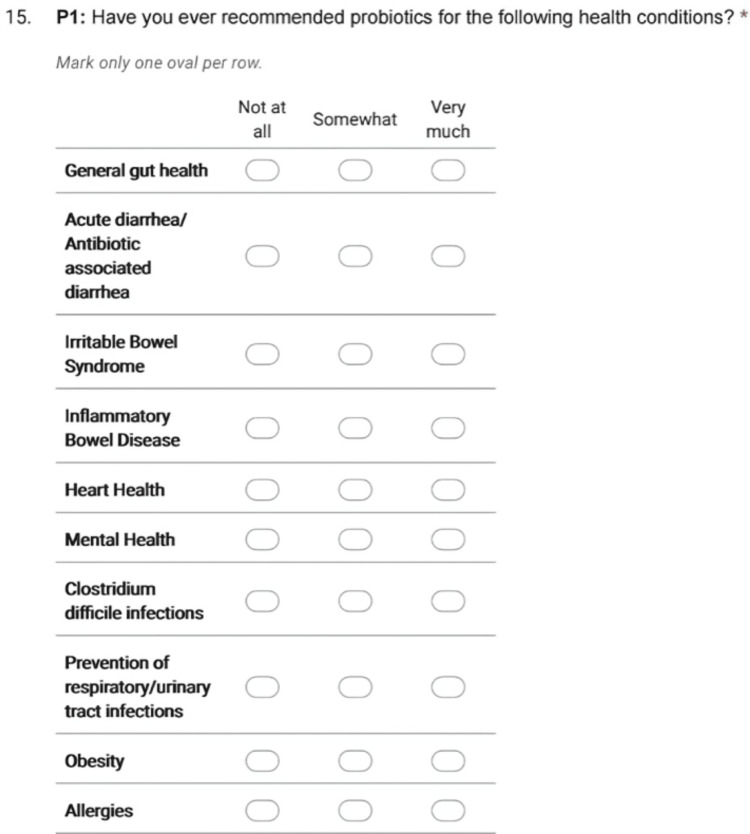
Questionnaire

**Figure 10:**
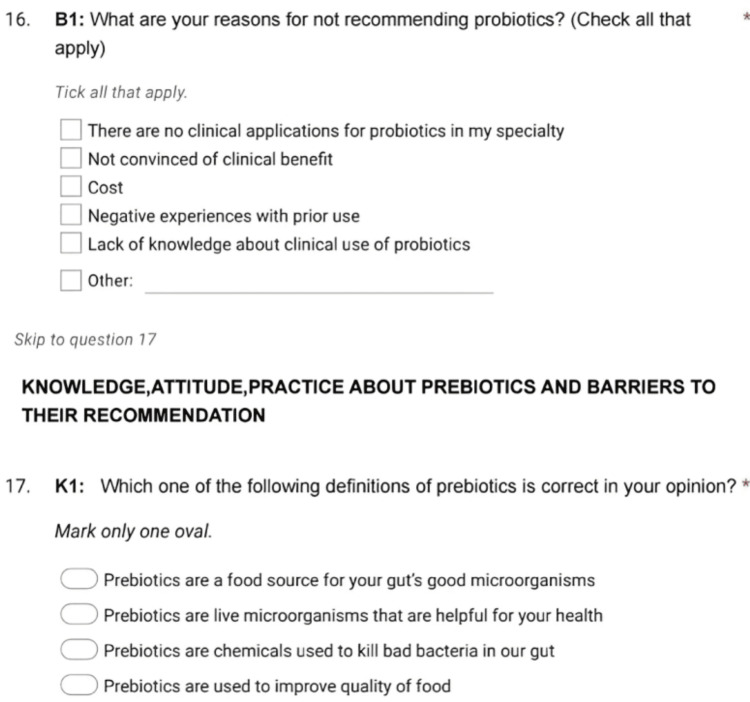
Questionnaire

**Figure 11:**
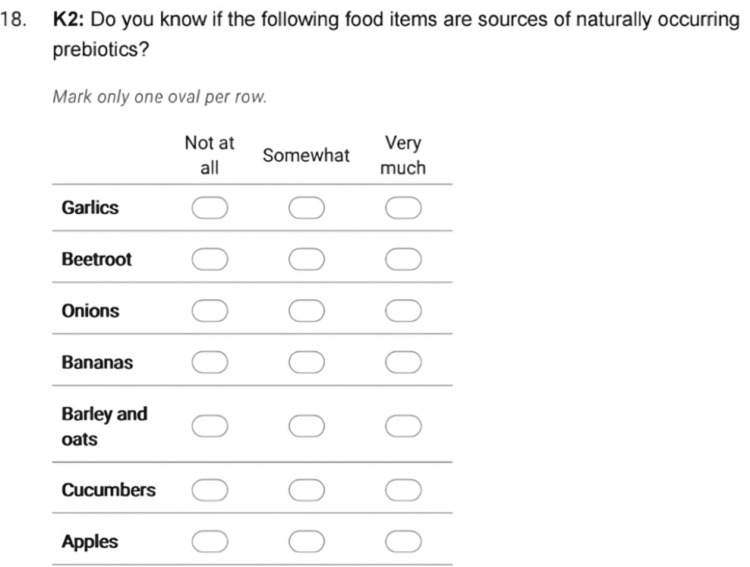
Questionnaire

**Figure 12:**
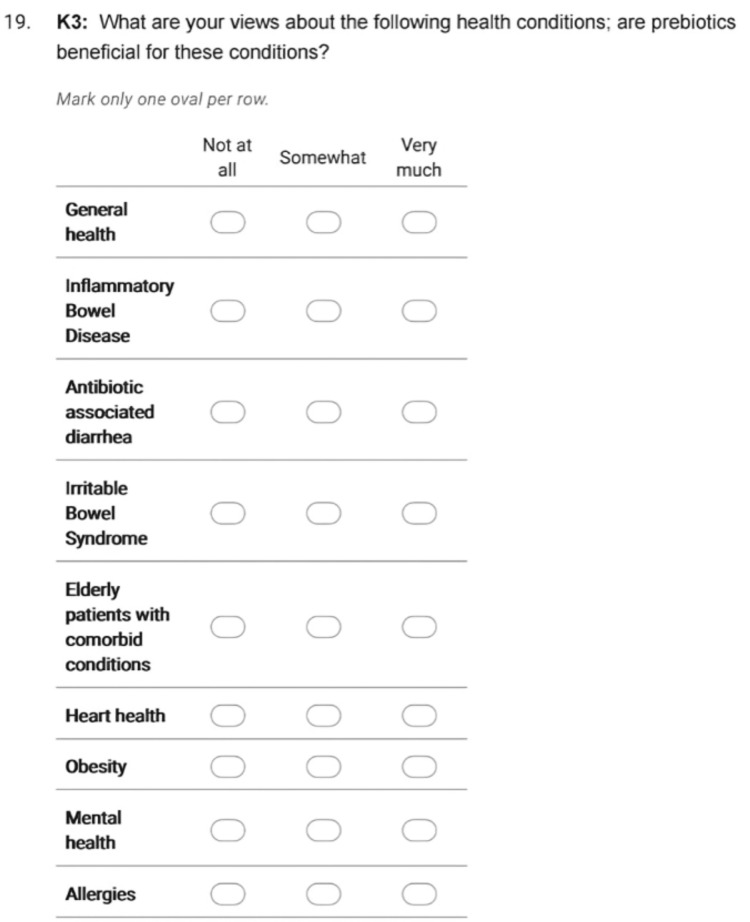
Questionnaire

**Figure 13:**
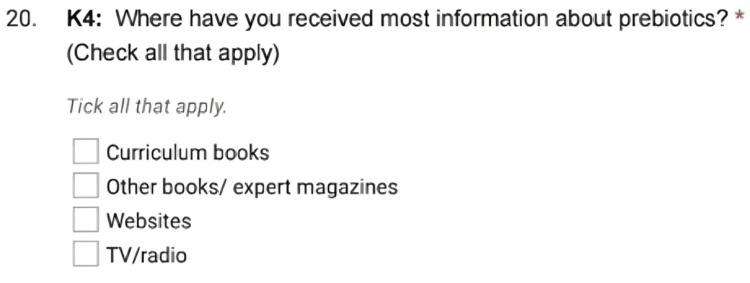
Questionnaire

**Figure 14:**
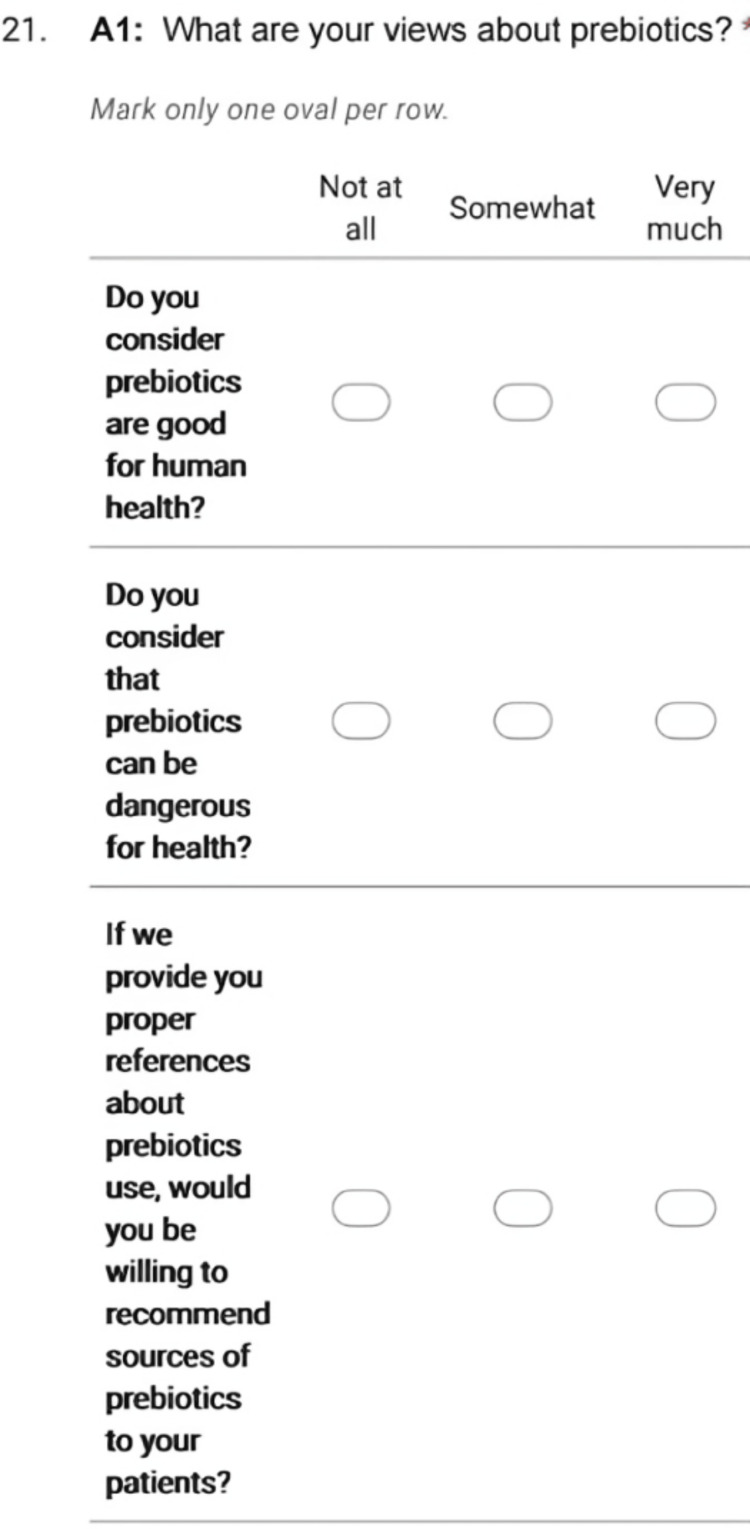
Questionnaire

**Figure 15:**
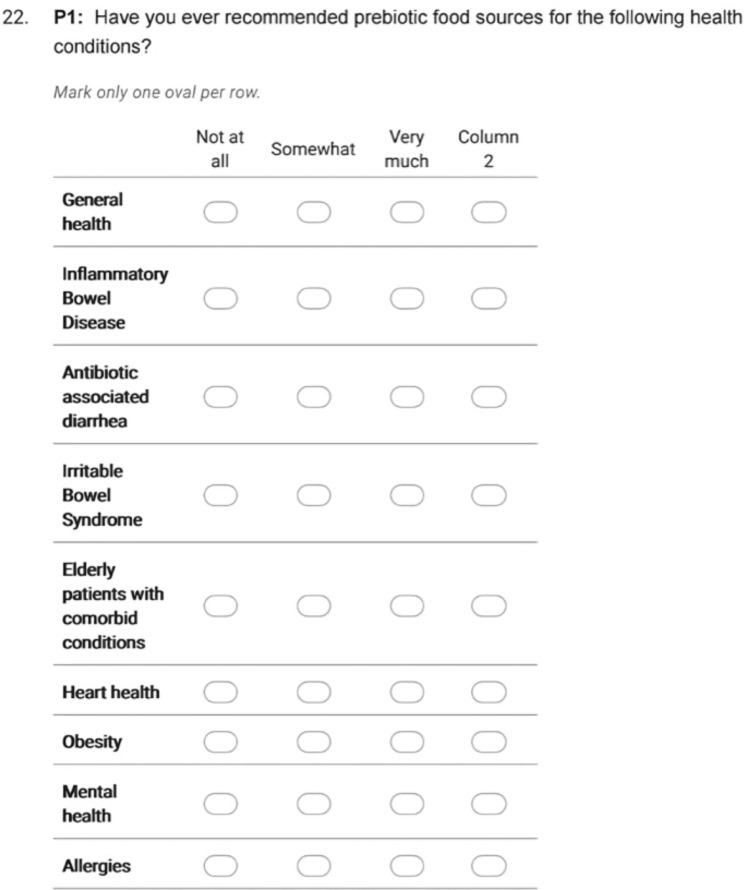
Questionnaire

**Figure 16:**
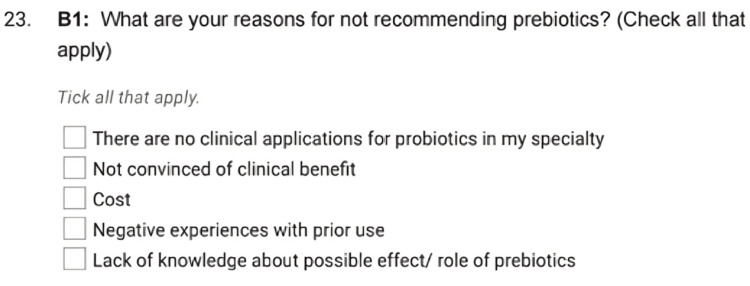
Questionnaire

## Appendices

**Table 4:** Supplementary Table 1, the Questionnaire used to assess the participants' knowledge, attitude, and practice regarding probiotics

Demographic data			
What is your gender? Male Female other			
What is your age?			
What is your marital status? Married unmarried			
What is your professional position? Medical student Doctor			
What is your patient population?(Check all that apply) Pediatric ( upto 17 years old) Adult ( 18-64 years old) Geriatric ( 65 years and above)			
What is your work experience? (Years in practice, for doctors and nursing staff) Student 1-4 years 5-9 years 10-14 years 15 years or more			
PROBIOTICS : KNOWLEDGE, ATTITUDE, PRACTICE ABOUT PROBIOTICS AND BARRIERS TO THEIR RECOMMENDATION			
K1: Which one of the following definitions of probiotics is correct in your opinion?			
Probiotics are live organisms (mostly bacteria) that are helpful for your health when you eat them.			1
Probiotics are diet products for the good bacteria in your body.			0
Probiotics are natural antibiotics.			0
Probiotics are chemicals to help kill bacteria on fruits and vegetables.			0
Probiotics are substances that make food taste sweeter.			0
K2: Are you familiar with the following commercially available probiotic drug brands?	Not at all	Somewhat	Very much
Enterogermina, oral suspensions by Sanofi Aventis	0	1	2
Enflor, sachets and capsules by Martin Dow Pharmaceuticals	0	1	2
Actiflor, sachets by Pharmevo	0	1	2
K3: What are your views about the following health conditions; are probiotics beneficial for these conditions?	Not at all	somewhat	Very much
General gut health	0	1	2
Acute diarrhea/ Antibiotic associated diarrhea	0	1	2
Irritable Bowel Syndrome	0	1	2
Inflammatory Bowel Disease	0	1	2
Heart Health	0	1	2
Mental Health	0	1	2
Clostridium difficile infections	0	1	2
Prevention of Respiratory/ Urinary tract infections	0	1	2
Obesity	0	1	2
Allergies	0	1	2
K4: Do any strains that belong to the below listed species of microorganisms belong to probiotics? ( Check all that apply)			
Lactobacillus acidophilus	1		
Bifidobacterium bifidum	1		
Mycobacterium avium	0		
Escherichia coli	1		
Lactobacillus rhamnosus	1		
Bacillus subtilis	1		
Enterococcus faecium	1		
K5: Choose all the statements from the following that you believe are true:			
The only probiotics that work are tablets, powders or capsules	0		
Probiotic bacteria need to be alive organisms	1		
Probiotics should be taken before a meal	1		
For a beneficial effect it is necessary to take probiotics for a long period of time as they disappear from the gut after 2 weeks	1		
K6: Where have you received most information about probiotics? (Check all that apply) Curriculum books Other books or expert magazines Websites Pharmacy TV/radio other			
				A1: What are your views about probiotics?	Not at all	somewhat	Very much
Do you consider probiotics are good for human health?	0	1	2
Do you consider that probiotics can be dangerous for health?	0	1	2
If we provide you proper reference about probiotics use, would you be willing to recommend probiotics to patients?	0	1	2
P1: Have you ever recommended probiotics for the following health conditions?	Not at all	Somewhat	Very much
General gut health	0	1	2
Acute diarrhea/ Antibiotic associated diarrhea	0	1	2
Irritable Bowel Syndrome	0	1	2
Inflammatory Bowel Disease	0	1	2
Heart Health	0	1	2
Mental Health	0	1	2
Clostridium difficile infections	0	1	2
Prevention of respiratory/urinary tract infections	0	1	2
Obesity	0	1	2
Allergies	0	1	2
B1: What are your reasons for not recommending probiotics? (Check all that apply) There are no clinical applications for probiotics in my specialty Not convinced of clinical benefit Cost Negative experiences with prior use Lack of knowledge about clinical use of probiotics Other			
				PREBIOTICS : KNOWLEDGE,ATTITUDE,PRACTICE ABOUT PREBIOTICS AND BARRIERS TO THEIR RECOMMENDATION			
K1: Which one of the following definitions of prebiotics is correct in your opinion?			
Prebiotics are a food source for your gut’s good microorganisms	1		
Prebiotics are live microorganisms that are helpful for your health	0		
Prebiotics are chemicals used to kill bad bacteria in our gut	0		
Prebiotics are used to improve quality of food	0		
K2: Do you know if the following food items are sources of naturally occurring prebiotics?	Not at all	Somewhat	Very much
Garlics	0	1	2
Beetroot	0	1	2
Onions	0	1	2
Bananas	0	1	2
Barley and oats	0	1	2
Cucumbers	0	1	2
Apples	0	1	2
K3: What are your views about the following health conditions; are prebiotics beneficial for these conditions?	Not at all	Somewhat	Very much
General health	0	1	2
Inflammatory Bowel Disease	0	1	2
Antibiotic associated diarrhea	0	1	2
Irritable Bowel Syndrome	0	1	2
Elderly patients with comorbid conditions	0	1	2
Heart health	0	1	2
Obesity	0	1	2
Mental health	0	1	2
Allergies	0	1	2
K4: Where have you received most information about prebiotics? (Check all that apply) Curriculum books Other books or expert magazines Websites Pharmacy TV/radio other			
				A1: What are your views about prebiotics?	Not at all	Somewhat	Very much
Do you consider prebiotics are good for human health?	0	1	2
Do you consider that prebiotics can be dangerous for health?	2	1	0
If we provide you proper references about prebiotics use, would you be willing to recommend sources of prebiotics to your patients?	0	1	2
P1: Have you ever recommended prebiotic food sources for the following health conditions?	Not at all	Somewhat	Very much
General health	0	1	2
Inflammatory Bowel Disease	0	1	2
Antibiotic associated diarrhea	0	1	2
Irritable Bowel Syndrome	0	1	2
Elderly patients with comorbid conditions	0	1	2
Heart health	0	1	2
Obesity	0	1	2
Mental health	0	1	2
Allergies	0	1	2
B1: What are your reasons for not recommending prebiotics? (Check all that apply) There are no clinical applications for probiotics in my specialty Not convinced of clinical benefit Cost Negative experiences with prior use Lack of knowledge about possible effect/ role of prebiotics			
				Probiotics			
Knowledge	Score range	Good score	Poor score
K1	0-1	1	0
K2	0-6	4-6	0-3
K3	0-20	11-20	0-10
K4	0-1	1	0
K5	0-1	1	0
Attitude A1	0-6	4-6	0-3
Practice P1	0-20	11-20	0-10
Prebiotics			
Knowledge	Score range	Good score	Poor score
K1	0-1	1	0
K2	0-14	8-14	0-7
K3	0-18	10-18	0-9
Attitude A1	0-6	4-6	0-3
Practice P1	0-18	10-18	0-9

## References

[1] Morelli L, Capurso L (2024). FAO/WHO guidelines on probiotics: 10 years later. J Clin Gastroenterol.

[2] (2024). Health and nutritional properties of probiotics in food including powder milk with live lactic acid bacteria. https://www.iqb.es/digestivo/pdfs/probioticos.pdf.

[3] Hill C, Guarner F, Reid G (2024). The International Scientific Association for Probiotics and Prebiotics consensus statement on the scope and appropriate use of the term probiotic. Nat Rev Gastroenterol Hepatol.

[4] Guarner F, Khan A (2024). World Gastroenterology Organisation Practice Guideline: Probiotics and Prebiotics - May 2008. S Afr Gastroenterol Rev.

[5] Gibson GR, Hutkins R, Sanders ME (2024). Expert consensus document: The International Scientific Association for Probiotics and Prebiotics (ISAPP) consensus statement on the definition and scope of prebiotics. Nat Rev Gastroenterol Hepatol.

[6] Pineiro M, Asp NG, Reid G, Macfarlane S, Morelli L, Brunser O, Tuohy K (2024). FAO technical meeting on prebiotics. J Clin Gastroenterol.

[7] Ambalam P, Raman M, Purama RK, Doble M (2024). Probiotics, prebiotics and colorectal cancer prevention. Best Pract Res Clin Gastroenterol.

[8] Kumar H, Salminen S, Verhagen H (2024). Novel probiotics and prebiotics: road to the market. Curr Opin Biotechnol.

[9] Markowiak P, Śliżewska K (2017). Effects of probiotics, prebiotics, and synbiotics on human health. Nutrients.

[10] Jenkins G, Mason P (2024). The role of prebiotics and probiotics in human health: a systematic review with a focus on gut and immune health. Food Nutr J.

[11] Kechagia M, Basoulis D, Konstantopoulou S, Dimitriadi D, Gyftopoulou K, Skarmoutsou N, Fakiri EM (2013). Health benefits of probiotics: a review. ISRN Nutr.

[12] de Vos WM, Tilg H, Van Hul M, Cani PD (2022). Gut microbiome and health: mechanistic insights. Gut.

[13] Dobson A, Cotter PD, Ross RP, Hill C (2012). Bacteriocin production: a probiotic trait?. Appl Environ Microbiol.

[14] Dai C, Zheng CQ, Jiang M, Ma XY, Jiang LJ (2013). Probiotics and irritable bowel syndrome. World J Gastroenterol.

[15] van Baarlen P, Wells JM, Kleerebezem M (2013). Regulation of intestinal homeostasis and immunity with probiotic lactobacilli. Trends Immunol.

[16] Arora M, Sharma S, Baldi A (2013). Comparative insight of regulatory guidelines for probiotics in USA, India and Malaysia: a critical review. Int J Biotechnol Wellness Ind.

[17] Gyawali R, Nwamaioha N, Fiagbor R, Zimmerman T (2019). The role of prebiotics in disease prevention and health promotion. Dietary Interventions in Gastrointestinal Diseases.

[18] Flesch AG, Poziomyck AK, Damin DC (2014). The therapeutic use of symbiotics. Arq Bras Cir Dig.

[19] Ababneh M, Elrashed N, Al-Azayzih A (2020). Evaluation of Jordanian healthcare providers' knowledge, attitudes, and practice patterns towards probiotics. Expert Rev Pharmacoecon Outcomes Res.

[20] Arshad MS, Saqlain M, Majeed A (2021). Cross-sectional study to assess the healthcare professionals' knowledge, attitude and practices about probiotics use in Pakistan. BMJ Open.

[21] Braun LA, Tiralongo E, Wilkinson JM, Spitzer O, Bailey M, Poole S, Dooley M (2010). Perceptions, use and attitudes of pharmacy customers on complementary medicines and pharmacy practice. BMC Complement Altern Med.

[22] Bridgman SL, Azad MB, Field CJ, Letourneau N, Johnston DW, Kaplan BJ, Kozyrskyj AL (2014). Maternal perspectives on the use of probiotics in infants: a cross-sectional survey. BMC Complement Altern Med.

[23] Owens C, Toone T, Steed-Ivie M (2014). A survey of dietary supplement knowledge, attitudes, and use in a rural population. J Nutr Food Sci.

[24] Fijan S, Frauwallner A, Varga L (2019). Health professionals' knowledge of probiotics: an international survey. Int J Environ Res Public Health.

[25] Soni R, Tank K, Jain N (2018). Knowledge, attitude and practice of health professionals about probiotic use in Ahmedabad, India. Nutr Food Sci.

[26] Amarauche CO (2015). Assessing the awareness and knowledge on the use of probiotics by healthcare professionals in Nigeria. J Young Pharm.

[27] O’Bryan CA, Pak D, Crandall PG, Lee SO, Ricke SC (2013). The role of prebiotics and probiotics in human health. J Prob Health.

